# Supplementary material for: Investigating food production‐associated DNA methylation changes in paleogenomes: Lack of consistent signals beyond technical noise
Source: Evol Appl. 2024 Jul 2;17(7):e13743. doi: 10.1111/eva.13743 (PMC11217591; doi:10.1111/eva.13743)
Supplement: Supplementary file 1 — Figure S1: Figure S2: Figure S3: Figure S4: Figure S5: Figure S6: Figure S7: Figure S8: Figure S9: [file EVA-17-e13743-s002.docx]

**Supplementary Information**

**Supplementary table legends**

**Supplementary Table 1.** Individual: individual genome ID as in the original article. Laboratory: the city where work has been conducted (or the senior author is based). Coverage: mean genome depth-of-coverage. Library: single or double-stranded library. Country: the country of origin of the ancient individual. Subsistence type: hunter-gatherer (HG) or Neolithic farmer (NF). Tissue: the tissue sampled for aDNA; bone or tooth. Total CpG positions: CpG positions out of 13 million (after excluding variable sites) that are covered by minimum 1 read. Total filtered >= 4: CpG positions out of 13 million (after excluding variable sites) that are covered by minimum 4 reads. Mean MS: mean methylation score (MS) per genome.

**Supplementary Table 2.** Number of genomes and mean methylation scores (MS) across those genomes per laboratory.

**Supplementary Table 3.** Pairwise Wilcoxon rank sum test results (*P*-values) over CGI, “shores3”, “shores5”, “shelf3”, “shelf5”, and “open sea” regions (Methods). Here we calculated the mean MS values for the respective regions, and performed comparisons using the values of all paleogenomes (n=34).

**Supplementary figures**

**
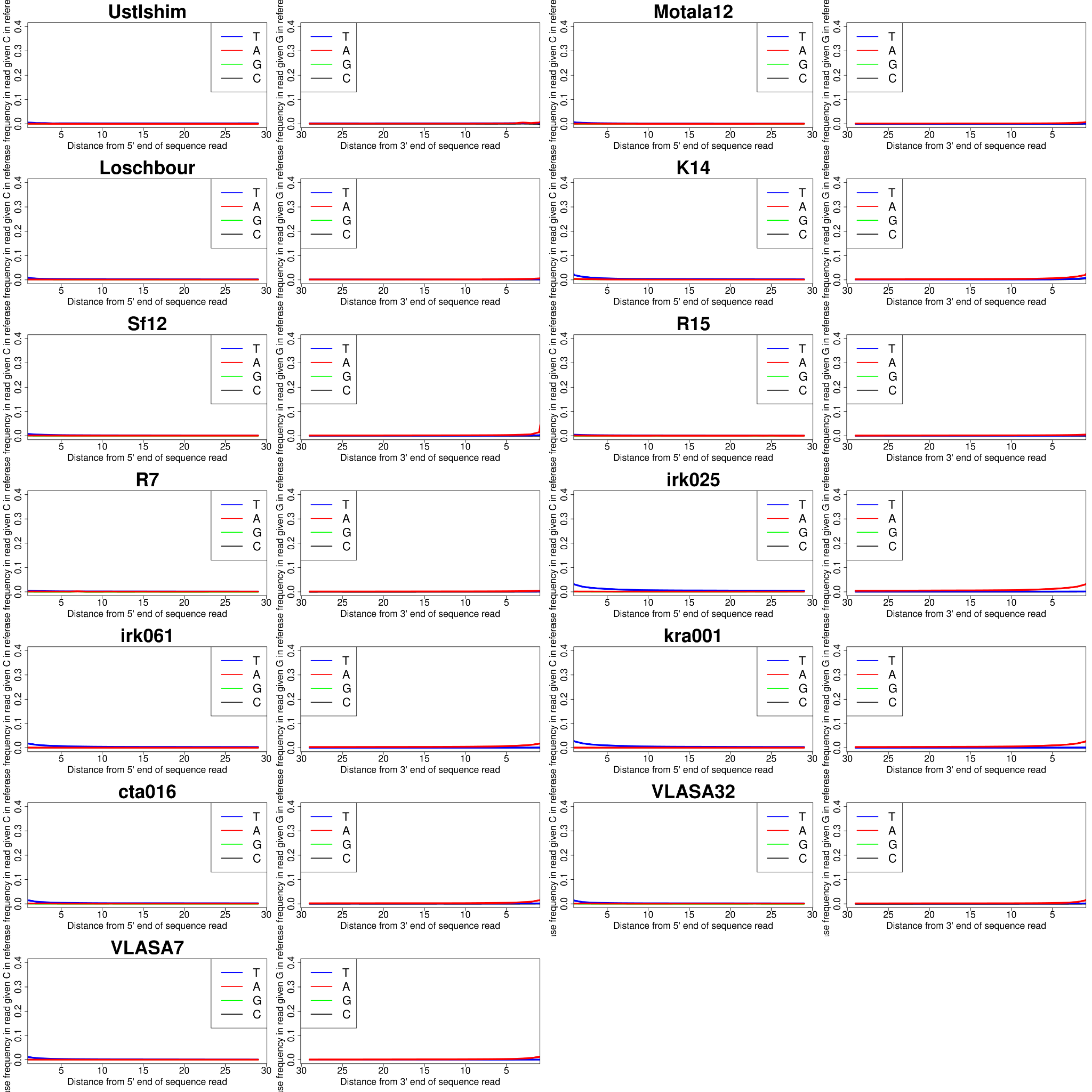
**

**Supplementary Figure 1.** Postmortem damage profiles of hunter-gatherer (HG) paleogenomes. The lack of excess C->T or G->A transition signals at 5’ and 3’ ends of reads, respectively, confirms the libraries were UDG-treated.

**
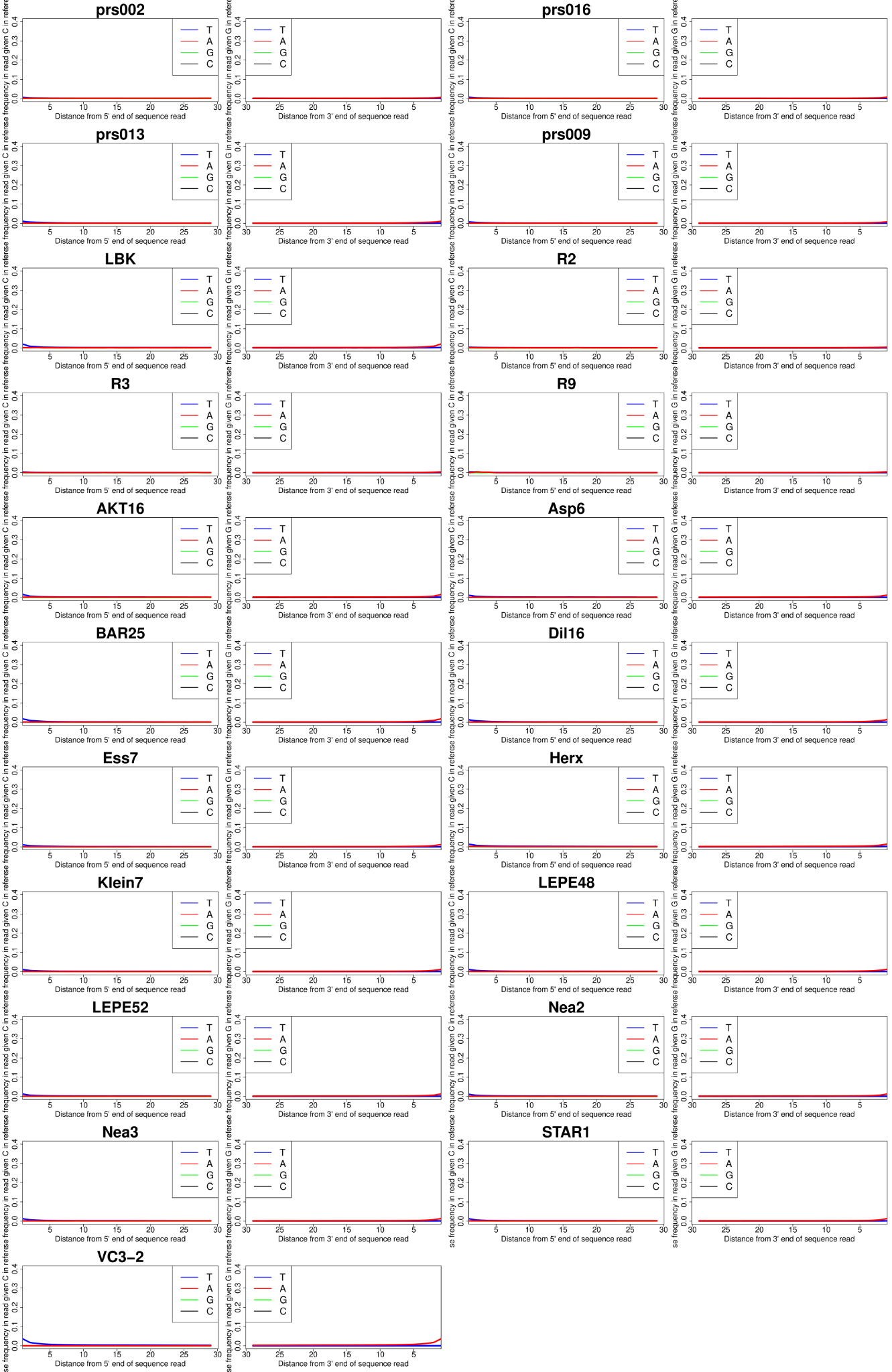
**

**Supplementary Figure 2.** Postmortem damage profiles of Neolithic farmer (NF) paleogenomes. The lack of excess C->T or G->A transition signals at 5’ and 3’ ends of reads, respectively, confirms the libraries were UDG-treated.

**
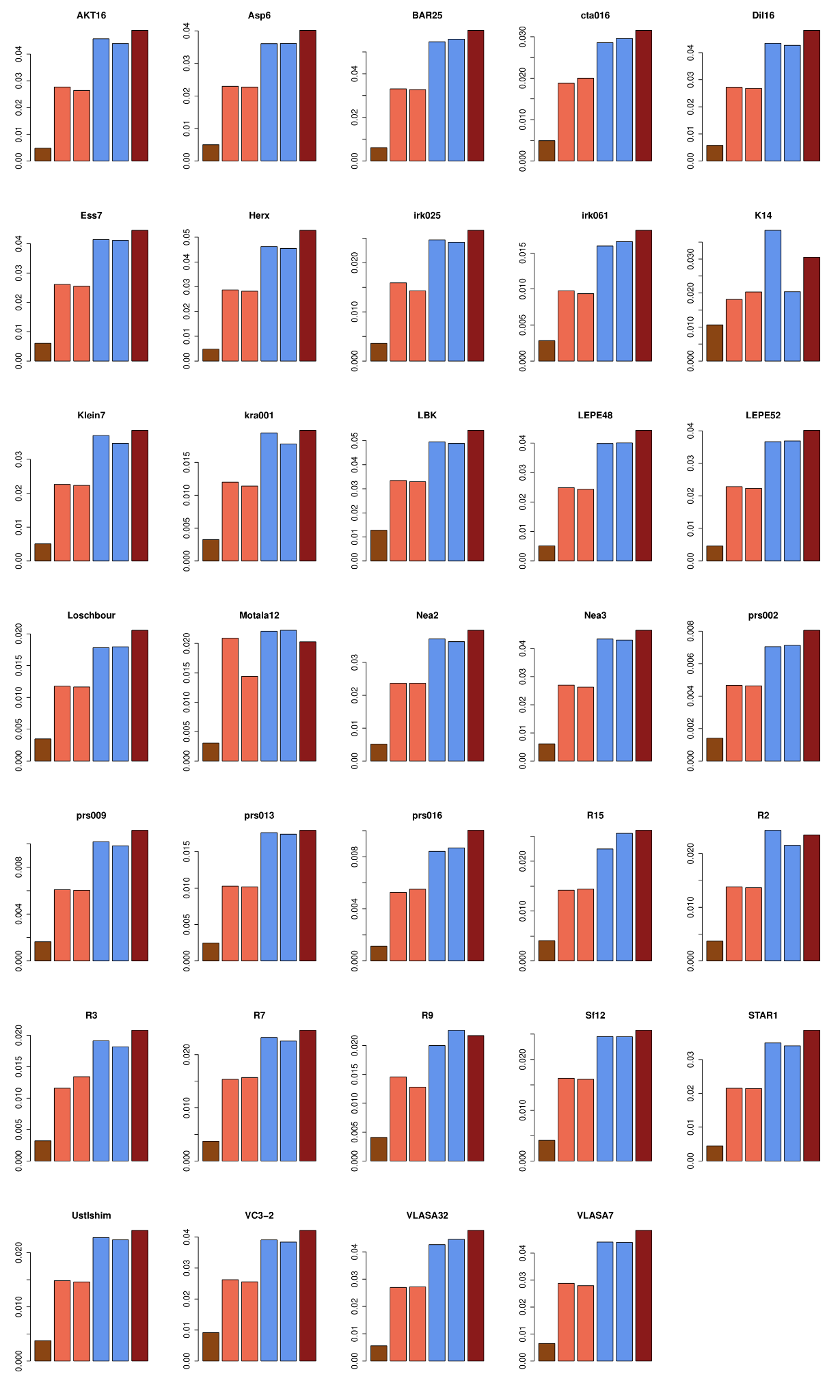
**

**Supplementary Figure 3.** The mean Methylation Scores (MS) on CpG islands (CGIs), “shelves”, “shores” and “open sea” areas of the genome per individual (continued in Supplementary Figure 4). The y-axes represent the mean MS and the x-axes indicate the genomic areas named above. The color brown represents CGIs, coral represents “shores”, blue indicates “shelves” and red represents “open sea” areas.


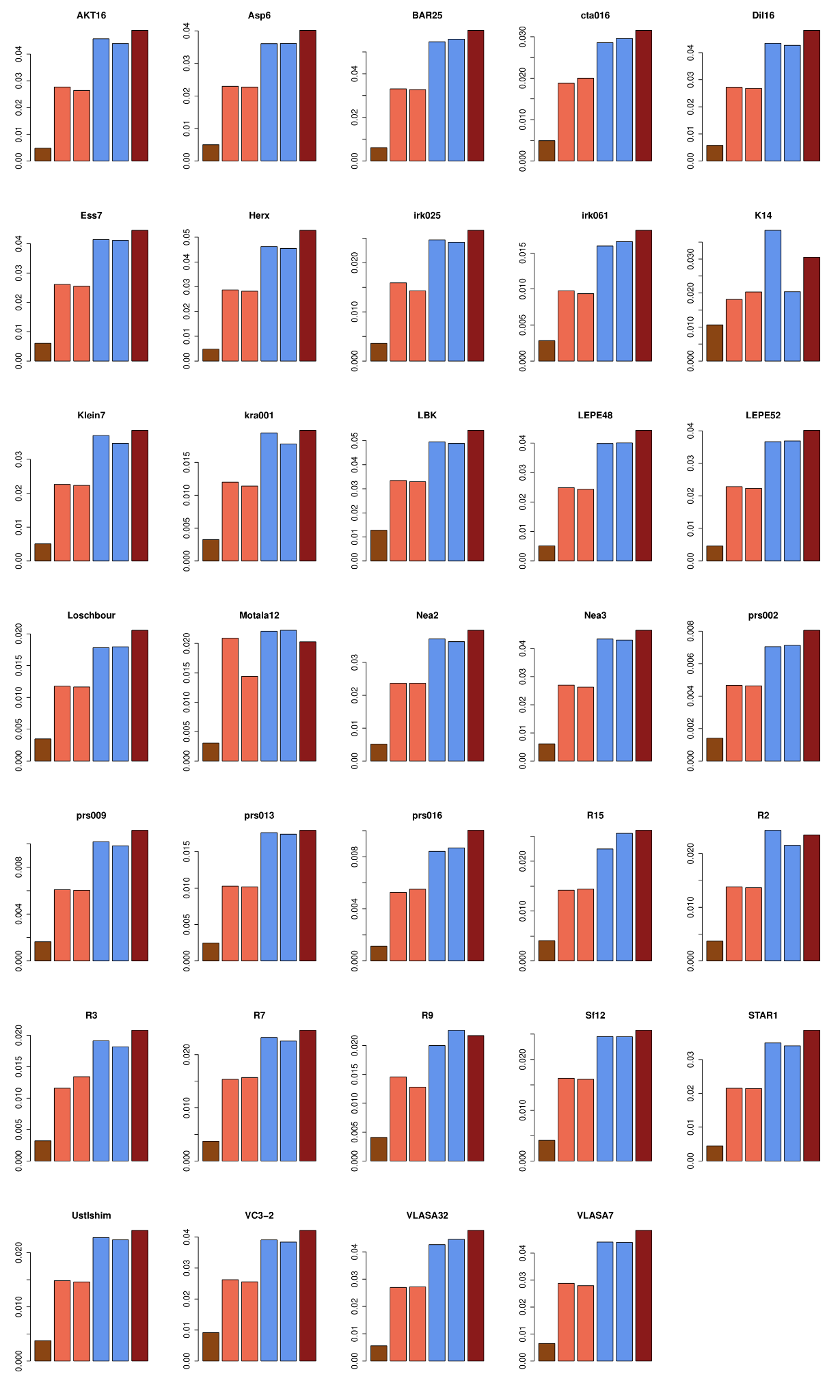


**Supplementary Figure 4.** The mean Methylation Scores (MS) on CpG islands (CGIs), “shelves”, “shores” and “open sea” areas of the genome per individual (continued from Supplementary Figure 3). The y-axes represent the mean MS and the x-axes indicate the genomic areas named above. The color brown represents CGIs, coral represents “shores”, blue indicates “shelves” and red represents “open sea” areas.


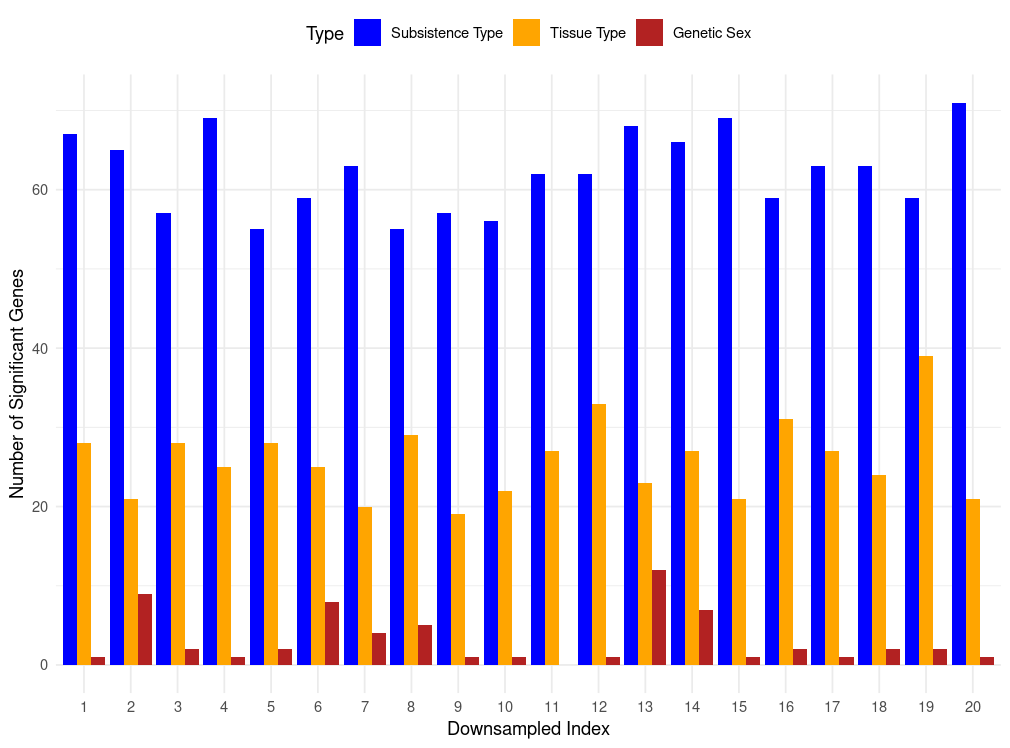


**Supplementary Figure 5.** The number of significant genes across the 20 replicate downsampled datasets. The x-axis represents the index of the replicate and the y-axis shows the number of significant genes (at ANOVA *P*<0.05 after BH-correction). The number of genes showing a subsistence type effect are colored as blue, tissue type effect as orange, and the genetic sex effect as red.


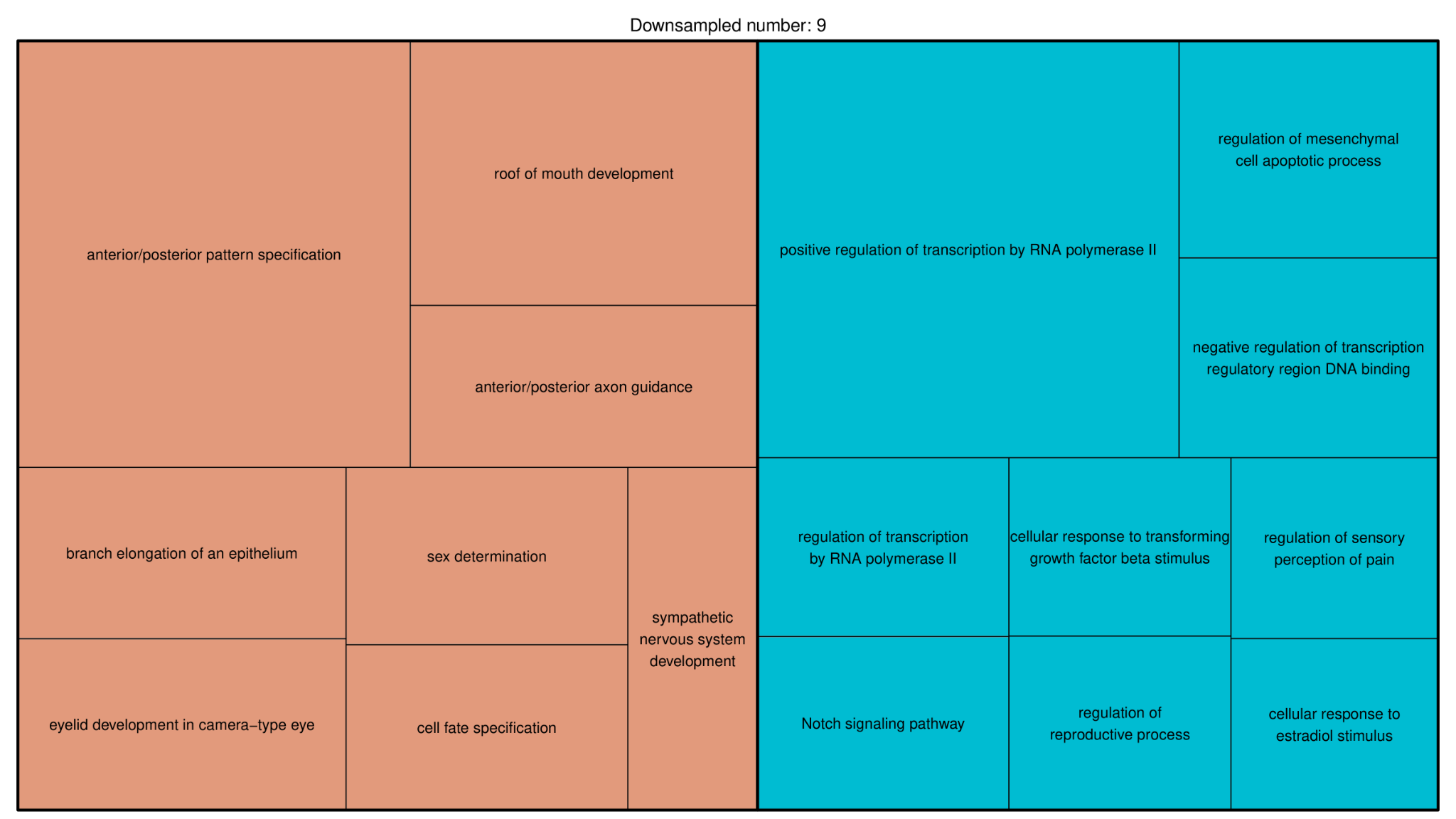


**Supplementary Figure 6.** Gene Ontology enrichment analysis results on subsampled dataset no. 9 for genes nominally significant for “subsistence type” in the linear mixed effect models with “individual” as random effect. Developmental processes are grouped on the left side (brown). Regulation-related processes are grouped on the right side (blue).


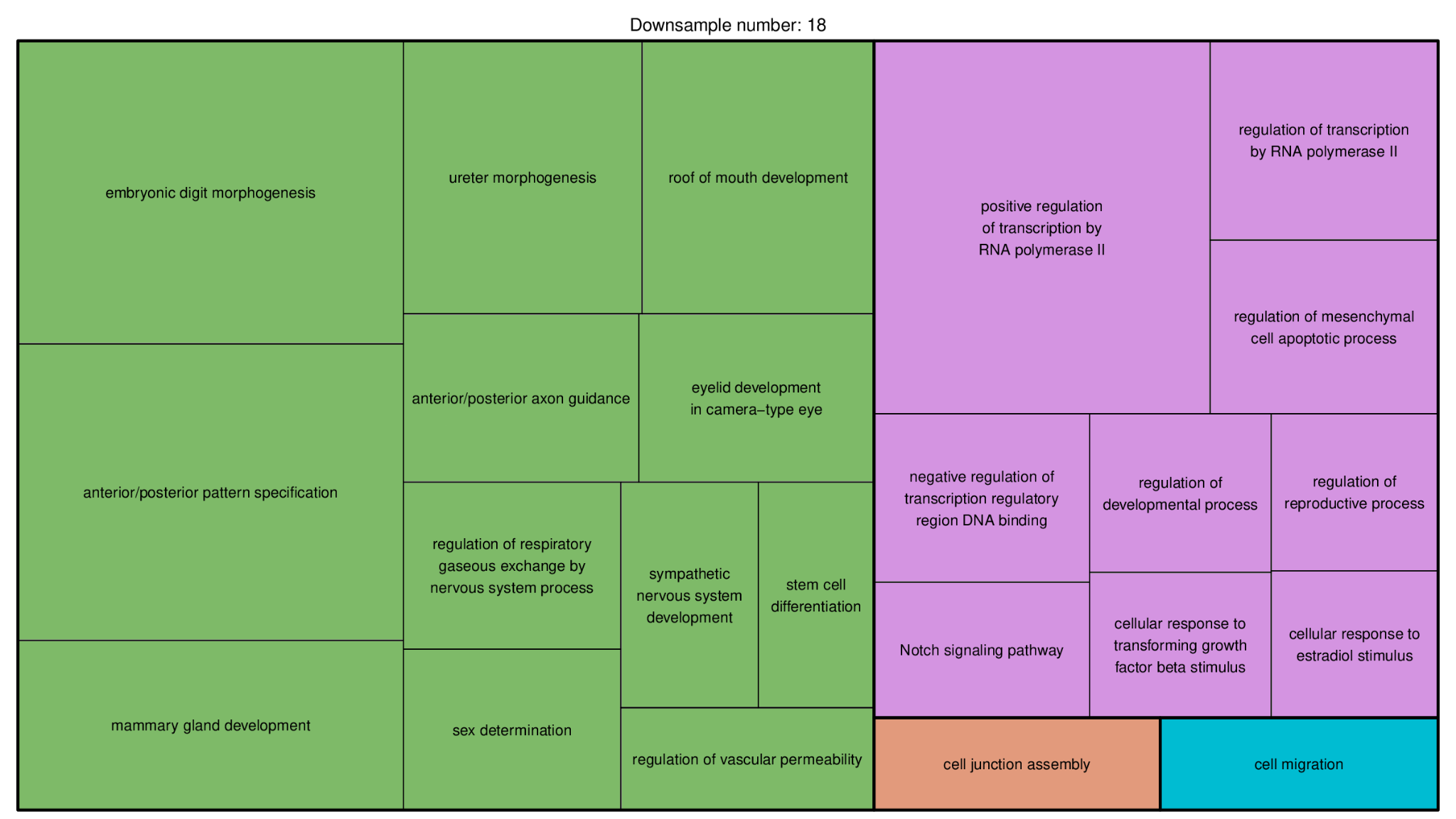


**Supplementary Figure 7.** Gene Ontology enrichment analysis results on subsampled dataset no. 18 for genes nominally significant for “subsistence type” in the linear mixed effect models with “individual” as random effect. Developmental processes are grouped on the left side (green). Regulation-related processes are grouped on the upper right side (purple).

**
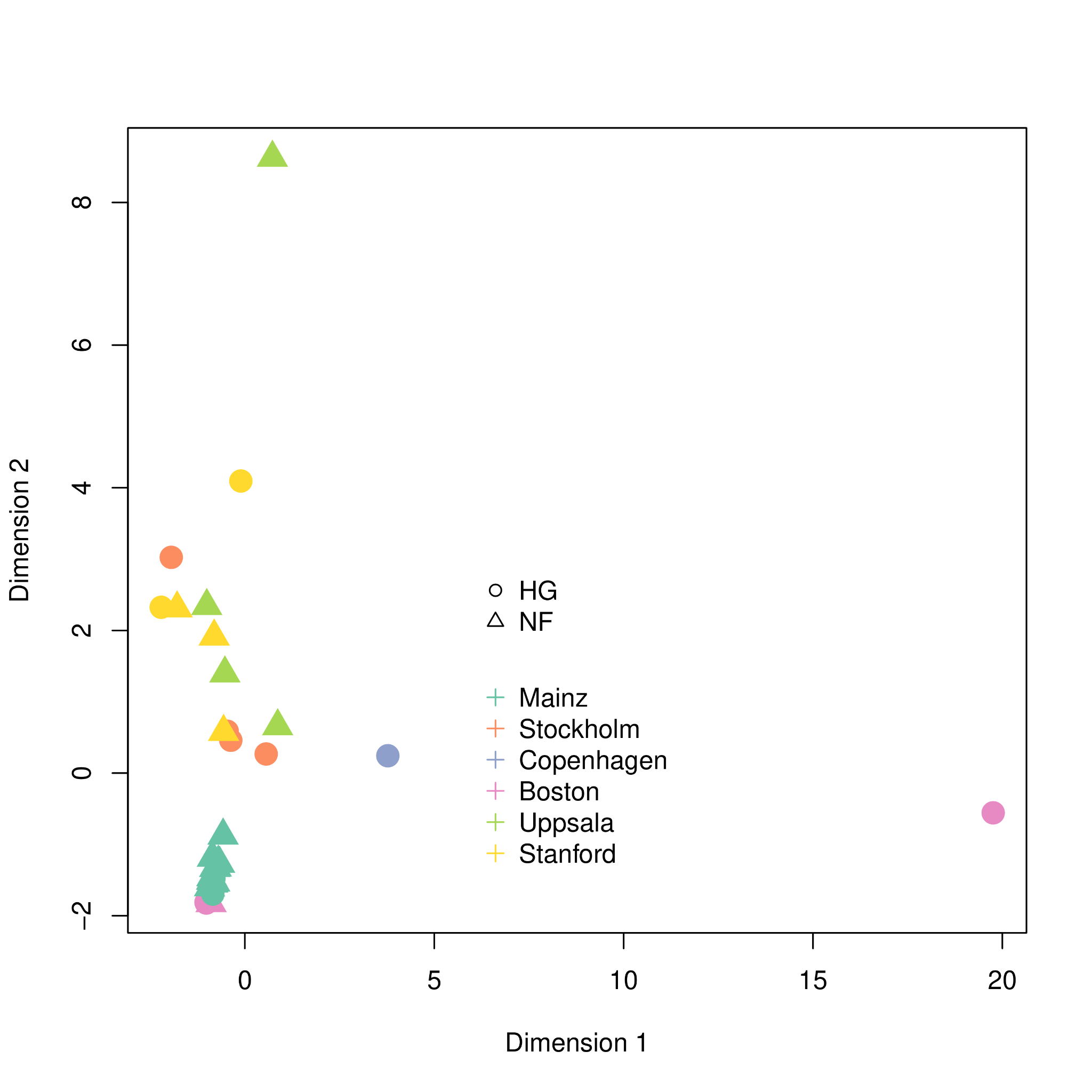
**

**Supplementary Figure 8.** Multidimensional scaling of the individuals by gene-averaged MS values. Ancient genomes representing hunter-gatherers (HG) are indicated by the circles and those representing Neolithic farmers (NF) by the triangles. Coloring of the illustration corresponds to the laboratory-of-origin of the samples. There are 6 different laboratories of which selected samples are produced from: Mainz (dark green), Stockholm (orange), Copenhagen (blue), Boston (pink), Uppsala (green), and Stanford (yellow). The Motala12 genome is on the bottom right and stands as an extreme outlier. The K14 genome the blue circle also shifted to the right of the main cluster.


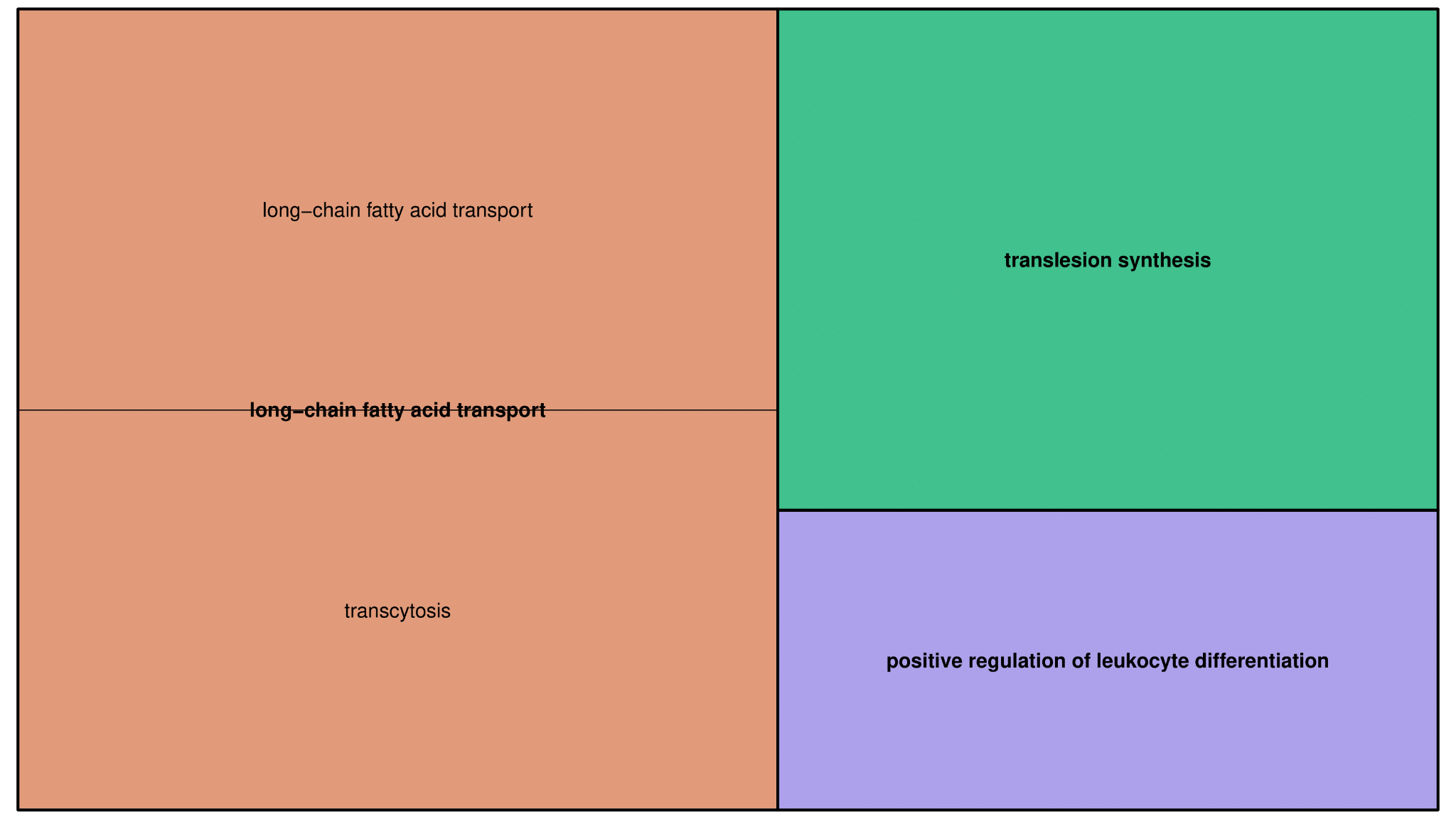


**Supplementary Figure 9.** Gene Ontology enrichment analysis results (read count per CpG position >= 10) for genes nominally significant for “subsistence type” in the linear mixed effect models with “individual” as random effect.

**Supplementary Text**

We reanalyzed the dataset from the beginning by filtering the number of reads being greater than or equal to 10. The number of individuals that had a sufficient amount of CpG positions per genome (>1000) reduced from 34 to 20 in this case. We normalized the data using random subsampling as in the main analyses, 20 times independently. We then calculated the mean MS per gene across the 20 replicate datasets. The number of genes included in the analysis was total 9880 (mean=3319 and median=2195 per individual with minimum 1 position observed).

We used our first model (deamination ~ subsistence type + tissue type + genetic sex + Error(individual) to calculate ANOVA and found 303, 278, and 69 significant genes at nominal *P*<0.05 for subsistence-type, tissue type and genetic sex, respectively. After the BH correction, these numbers reduced to 44, 37 and 3 genes. Note that the deamination values here are the mean across the 20 replicate datasets.

We then carried out GO Biological Process enrichment analysis using the same methodology as previously reported. We found similar GO categories to our previous results (minimum 4 reads) without correcting the *P*-values (Fisher’s exact test *P*<0.01) (Supplementary Figure 9), but no result was significant after the *P*-value adjustment using BH-correction.

Overall, increasing the read count per CpG position did not change the outcome of not observing epigenetic differences related to lifestyle in the current setting.
